# Supplementary material for: Maternal Immune Cell and Cytokine Profiles to Predict Cardiovascular Risk Six Months after Preeclampsia
Source: J Clin Med. 2022 Jul 19;11(14):4185. doi: 10.3390/jcm11144185 (PMC9317739; doi:10.3390/jcm11144185)
Supplement: Supplementary file 1 [file jcm-11-04185-s001.zip › jcm-1793064-supplementary.pdf]

Supplementary Materials

Table S1. Antibody Panel.

Figure S1. Inter- and Intra- Assay Consistency.

Figure S2. Flow cytometry gating strategy.

Table S2. Tabular summary of quantified cell types at delivery, 3- and 6-months postpartum.

Table S1. Antibody Panel.

Excitation source	Filter	Antibody	Distributor and Catalogue No.
Violet Laser	780/60 BP	BV786 Mouse Anti-Human CD45	BD Biosciences; 563716
	710/50 BP	BV711 Mouse Anti-Human CD14	BD Biosciences; 563373
	660/20 BP	BV650 Mouse Anti-Human CD25	BD Biosciences; 563719
	610/20 BP	BV605 Mouse Anti-Human CD16	BD Biosciences; 563172
	525/50 BP	Live/Dead® Fixable Dead Cells Stain Kit	Thermo Fischer Scientific; L34957; L34966
	450/40 BP	V450 Mouse Anti-Human CD3	BD Biosciences; 560365
Blue Laser	710/50 BP	BB700 Mouse Anti-Human CD11b	BD Biosciences; 742210
	530/30 BP	Alexa Fluor® 488 Mouse anti-Human FoxP3	BD Biosciences; 560047
Y-G Laser	780/60 BP	CD4 Monoclonal Antibody (RPA-T4), PE-Cyanine7, eBioscience™	Thermo Fischer Scientific; 25-0049-42
	710/50 BP	CD4 Mooclonal Antibody (S3.5), PE-Cyanine5.5, Invitrogen	Thermo Fischer Scientific; MHCD0418
	610/20 BP	PE-CF594 Mouse Anti-Human HLA-DR	BD Biosciences; 562304
	586/15 BP	PE Mouse Anti-Human CD56	BD Biosciences; 555516
Red Laser	780/60 BP	APC-Cy™7 Mouse Anti-Human CD19	BD Biosciences; 561743, 557791
	670/14 BP	APC Mouse Anti-Human CD69	BD Biosciences; 555533
UV Laser	379/28 BP	BUV395 Mouse Anti-Human CD8	BD Biosciences; 563795
	515/30 BP	BUV496 Mouse Anti-Human CD8	BD Biosciences; 612942

Figure S1. Inter- and Intra- Assay Consistency.

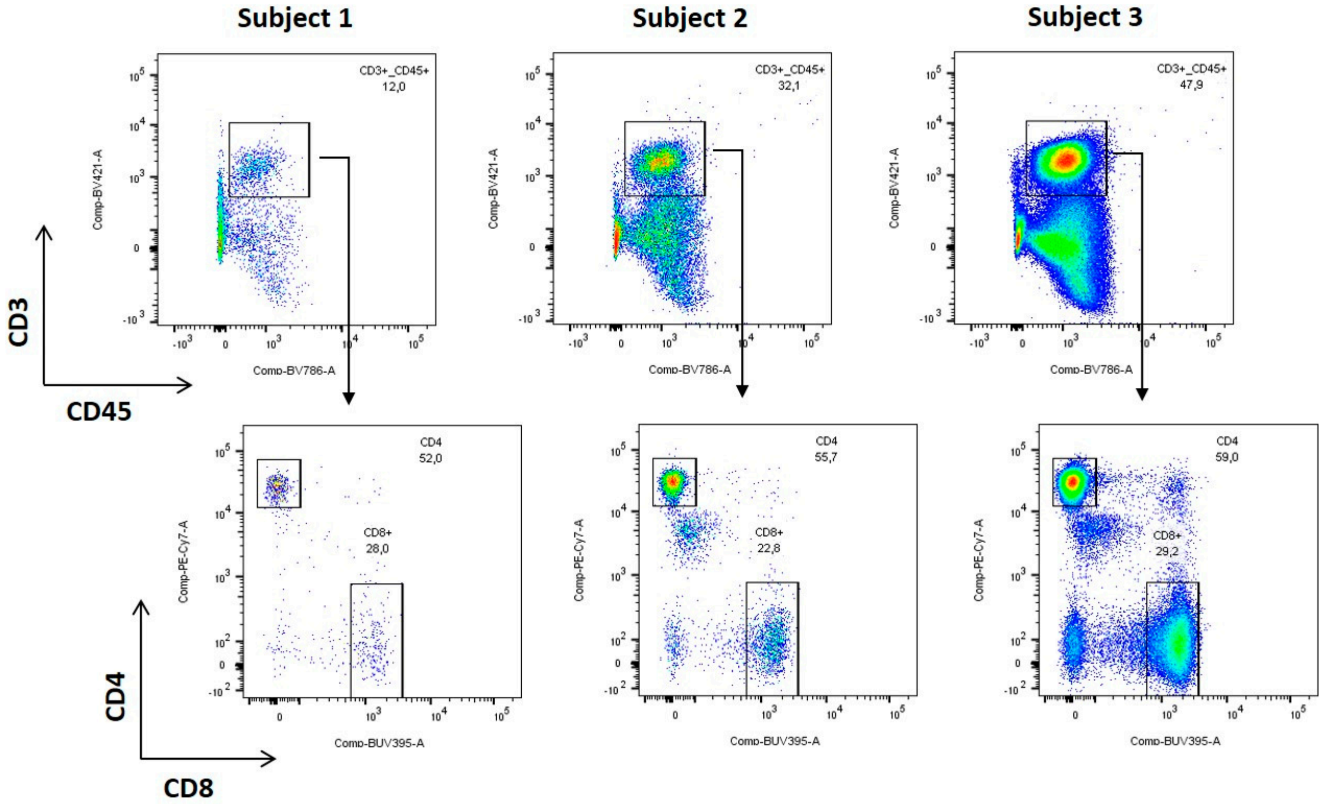

The final FACS panel showed very good consistency with gating for major immune cell populations within and between samples tested on the same day, as well as between different acquisition days. Inter-assay consistency is shown in the Figure below. Intra-assay consistency testing was similar (data not shown). Similar results were seen across all cell populations.

Figure S2. Flow cytometry gating strategy.

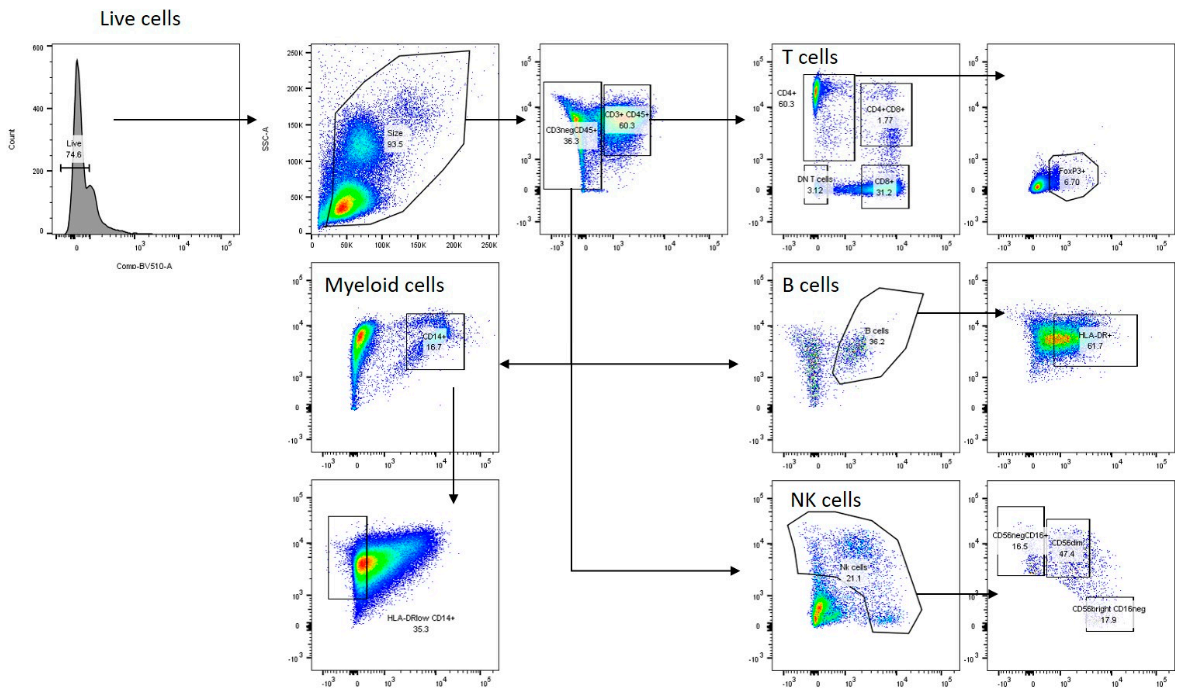

A traditional gating strategy was used to identify T cells, B cells, NK cells and Myeloid cells. A traditional gating strategy was also used to identify CD25+ and CD69+ activated T cells (figure/data not shown).

Table S2. Tabular summary of quantified cell types at delivery, 3- and 6-months postpartum.

	**Percentage Cell Populations [a], Mean ± Standard Deviation**								
	**At Delivery, *n* = 15**			**At 3 Months Postpartum, *n* = 16**			**At 6 Months Postpartum, *n* = 28**		
**Cell type**	**Low Risk N = 5**	**High Risk N = 10**	***p*-Value**	**Low Risk N = 7**	**High Risk N = 9**	***p*-Value**	**Low Risk N = 14**	**High Risk N = 14**	***p*-Value**
				T Cells					
CD3$^+$CD45$^+$ T cells	54.0 ± 8.4	47.2 ± 13.1	0.316	54.4 ± 10.5	56.9 ± 6.8	0.577	48.8 ± 9.0	50.6 ± 13.4	0.672
└ CD4$^+$ T cells	60.5 ± 12.9	61.9 ± 11.1	0.839	64.1 ± 11.8	56.5 ± 11.9	0.223	64.6 ± 12.2	58.9 ± 9.2	0.171
└ FoxP3$^+$ regulatory T cells	6.9 ± 2.5	5.6 ± 2.2	0.321	8.1 ± 1.8	6.1 ± 1.1	0.014*	7.0 ± 2.6	5.8 ± 1.8	0.192
└ CD8$^+$ T cells	28.3 ± 9.9	28.2 ± 10.5	0.977	22.1 ± 8.4	33.5 ± 8.7	0.0204*	23.5 ± 9.6	28.8 ± 8.5	0.137
└ CD4$^+$CD8$^+$ (double positive) T cells	1.3 ± 0.3	1.3 ± 0.6	0.964	1.7 ± 0.6	1.7 ± 0.4	0.843	1.2 ± 0.5	1.4 ± 0.5	0.368
└ CD4$^-$CD8$^-$ (double negative) T cells	5.5 ± 2.6	4.9 ± 2.8	0.681	7.3 ± 4.1	4.5 ± 2.2	0.0920	6.5 ± 3.1	7.0 ± 3.3	0.652
└ CD25$^+$ activated T cells	0.2 ± 0.1	0.2 ± 0.1	0.838	0.3 ± 0.2	0.3 ± 0.1	0.614	0.3 ± 0.2	0.2 ± 0.1	0.329
└ CD69$^+$ activated T cells	2.8 ± 1.2	2.5 ± 1.5	0.725	2.6 ± 1.4	2.2 ± 0.5	0.466	2.3 ± 1.1	1.9 ± 0.7	0.330
CD4$^+$:CD8$^+$ ratio	2.5 ± 1.2	2.7 ± 1.5	0.800	3.3 ± 1.3	1.9 ± 0.9	0.0243*	3.4 ± 1.9	2.3 ± 0.9	0.0595
				Non-T Cell Lymphocytes and myeloid cells					
CD3$^-$CD45$^+$ N	42.3 ± 8.2	48.1 ± 12.6	0.367	41.4 ± 9.4	40.5 ± 6.5	0.813	46.9 ± 8.7	43.1 ± 10.1	0.298
└ CD19$^+$ B cells	6.0 ± 3.5	7.2 ± 3.7	0.560	7.2 ± 2.9	7.8 ± 2.4	0.615	9.5 ± 9.0	8.4 ± 3.1	0.657
└HLA-DR$^+$ B cells	44.7 ± 25.7	41.3 ± 15.1	0.749	61.9 ± 19.8	74.1 ± 8.3	0.115	61.2 ± 16.8	62.2 ± 10.5	0.854
└ CD3$^-$CD45$^+$ NK cells	15.2 ± 5.5	8.7 ± 5.1	0.0401 *	13.9 ± 9.1	23.2 ± 10.3	0.0813	15.8 ± 11.2	13.9 ± 7.3	0.598
└ CD56^{bright}CD16$^-$ NK cells	11.3 ± 8.4	20.6 ± 24.6	0.432	12.0 ± 8.0	8.9 ± 6.9	0.426	12.9 ± 11.5	10.9 ± 12.8	0.663
└ CD56^{dim} NK cells	50.4 ± 5.0	38.4 ± 19.0	0.194	48.2 ± 11.0	43.6 ± 15.0	0.507	42.6 ± 12.4	38.6 ± 15.1	0.449
└ CD56$^-$CD16$^+$ NK cells	25.1 ± 9.2	28.7 ± 14.0	0.610	26.3 ± 9.0	37.1 ± 12.1	0.0683	29.7 ± 12.4	38.3 ± 17.6	0.149
└ CD14$^+$ myeloid cells	18.9 ± 8.9	17.5 ± 11.4	0.809	16.0 ± 10.0	15.0 ± 4.0	0.771	11.2 ± 7.6	9.4 ± 6.0	0.495
└ CD11b$^+$ monocytes	94.2 ± 2.4	94.2 ± 3.0	0.985	89.2 ± 11.0	95.2 ± 1.3	0.123	91.5 ± 6.4	89.5 ± 8.6	0.495
└ HLA DR$^+$ monocytes	6.5 ± 4.7	7.5 ± 7.7	0.803	8.9 ± 4.9	10.5 ± 3.0	0.420	6.3 ± 4.2	5.2 ± 4.1	0.490

* $p < 0.05$. [a] All cell populations are reported as frequency within the parent cell populations, except for CD3$^-$CD45$^+$/CD19$^+$ B cells which are reported as the frequency within the grandparent population (See gating strategy detailed in the supplementary materials for reference)
